# Topiramate inhibits adjuvant-induced chronic orofacial inflammatory allodynia in the rat

**DOI:** 10.3389/fphar.2024.1461355

**Published:** 2024-08-16

**Authors:** Violetta Mohos, Máté Harmat, Jozsef Kun, Tímea Aczél, Balázs Zoltán Zsidó, Tamás Kitka, Sándor Farkas, Erika Pintér, Zsuzsanna Helyes

**Affiliations:** ^1^ Department of Pharmacology and Pharmacotherapy, Medical School, University of Pécs, Pécs, Hungary; ^2^ Hungarian Centre for Genomics and Bioinformatics, Szentágothai Research Centre, University of Pécs, Pécs, Hungary; ^3^ National Laboratory for Drug Research and Development, Budapest, Hungary; ^4^ Uzsoki Cardiovascular Center Ltd., Budapest, Hungary; ^5^ Hungarian Research Network, PTE HUN-REN Chronic Pain Research Group, Budapest, Hungary; ^6^ PharmInVivo Ltd., Pécs, Hungary

**Keywords:** topiramate, sumatriptan, orofacial pain, trigeminal activation, inflammatory pain, animal model, complete Freund’s adjuvant, mechanical allodynia

## Abstract

Chronic orofacial pain disorders are common debilitating conditions, affecting the trigeminal system. Its underlying pathophysiological mechanisms are still unclear and the therapy is often unsatisfactory, therefore, preclinical models are crucial to identify the key mediators and novel treatment options. Complete Freund’s adjuvant (CFA)-induced orofacial inflammatory allodynia/hyperalgesia is commonly used in rodents, but it has not been validated with currently used drugs. Here we tested the effects of the adjuvant analgesic/antiepileptic voltage-gated Na^+^ channel blocker complex mechanism of action topiramate in comparison with the gold standard antimigraine serotonin 5-HT1B/D receptor agonist sumatriptan in this model. CFA was injected subcutaneously into the right whisker pad of male Sprague-Dawley rats (250–300 g), then mechanonociceptive threshold values were investigated with von Frey filaments (3, 5, and 7 days after CFA injection). Effects of topiramate (30 mg/kg *per os*) and sumatriptan (1 mg/kg *subcutaneous*) on the adjuvant-induced chronic inflammatory orofacial allodynia were investigated 60, 120, and 180 min after the treatments each day. To determine the optimal concentration for drug effect analysis, we tested the effects of two different CFA-concentrations (1 and 0.5 mg/mL) on mechanonociceptive thresholds. Both concentrations of CFA induced a chronic orofacial allodynia in 60% of all rats. Although, higher CFA concentration induced greater allodynia, much more stable threshold reduction was observed with the lower CFA concentration: on day 3 the thresholds decreased from 18.30 g to approximately 11 g (low) and 5 g (high), respectively, however a slight increase was observed in the case of higher CFA concentration (on days 5, 7, and 11). In all investigation days, topiramate showed significant anti-allodynic effect comparing the pre and post drug dose and comparing the vehicle treated to the drug treated groups. Sumatriptan also caused a significant threshold increase compared to pre dose thresholds (day 3) and also showed a slight anti-allodynic effect compared to the vehicle-treated group (day 3 and 5). In the present study CFA-induced chronic orofacial allodynia was reversed by topiramate in rats validating the model with the adjuvant analgesic. Other than establishing a validated orofacial pain-related syndrome model in rats, new ways are opened for the repurposing of topiramate.

## 1 Introduction

Orofacial pain disorders (e.g., temporomandibular disorders, trigeminal neuralgia, facial migraine) are exhausting conditions affecting the trigeminal system and reducing the quality of life (Romero-Reyes and Uyanik, 2014; Rotpenpian and Yakkaphan, 2021). They are commonly caused by inflammation or injury (Tseng et al., 2012; Bista and Imlach, 2019), compression of the trigeminal nerve (Leal et al., 2014; Bista and Imlach, 2019), demyelinating diseases (Cruccu et al., 2009), neoplastic infiltration (Gottfried et al., 2007), or herpes virus infections (Bista and Imlach, 2019). In many cases, the etiology and the pathophysiological mechanisms are unclear, however it has been suggested that both peripheral and central dysfunctions may play crucial roles (Leal et al., 2014; Costa and Leite, 2015; Bista and Imlach, 2019; Gündüz et al., 2019). Since the pharmacotherapy of orofacial pain is often unsatisfactory, sensitization mechanisms need to be better understood to identify novel treatment options (Sessle, 2021; Romero-Reyes et al., 2023).

According to the most recent guidelines for trigeminal neuralgia the antiepileptic voltage-gated Na^+^ and Ca^2+^ channel blocker carbamazepine is considered as a first-line therapy (Bendtsen et al., 2019; Clark et al., 2020), remaining the only drug with this approved indication (Wu et al., 2017). Notably, several clinical studies were conducted on facial pain and later referred to as trigeminal neuralgia, but the quality of these trials underlying the efficacy of carbamazepine for this indication was questioned due to limited number of involved patients and inconsistent outcome reports (Martin and Forouzanfar, 2011; Wiffen et al., 2011). Furthermore, adverse effects were also reported to frequently occur (Wiffen et al., 2011). In carbamazepine-resistant trigeminal neuralgia patients topiramate was found to be effective in case studies (Zvartau-Hind et al., 2000; Solaro et al., 2001; Domingues et al., 2007) involving small group of patients. A meta-analysis comparing the efficacy of topiramate *versus* carbamazepine for trigeminal neuralgia treatment concluded that topiramate was superior, however the low quality of the underlying clinical trials was highlighted as a limitation (Romero-Reyes and Uyanik, 2014). Carbamazepine-resistant cases of trigeminal pain, high frequency of several adverse effects and poor quality of clinical trials reporting on carbamazepine or alternative therapeutical options necessitate more evidence on the efficacy of drugs against orofacial pain conditions.

It has been described that orofacial inflammation activates and sensitizes the trigeminal primary and secondary sensory neurons, which induces mechanical allodynia/hyperalgesia both in humans and animal models (Iwata et al., 2017). Animal models are crucial in the development of novel therapies, but none of them show all aspects of the disorders (Chou and Chen, 2018), therefore, there is a constant need for further improvement in this field (Laborc et al., 2020). CFA-induced inflammation and subsequent allodynia/hyperalgesia is commonly used to explore the mechanisms involved in acute or chronic pain conditions (Takeda et al., 2008; Magni et al., 2015; Takeda et al., 2018; McCarson and Fehrenbacher, 2021; Lin et al., 2022). In our previous study, a CFA-induced orofacial inflammatory allodynia model was set up to investigate transcriptomic changes in the trigeminal ganglion (TG) and the trigeminal nucleus caudalis (TNC). The detected alterations are associated with the onset and time course of peripheral and central sensitization, and they were reflected by similar changes in the peripheral blood mononuclear cells (Aczél et al., 2018; Aczél et al., 2020).

In animal models of trigeminal neuralgia, mainly carbamazepine (Chogtu et al., 2011; Hahm et al., 2012; Pineda-Farias et al., 2021; Baggio et al., 2024; Song et al., 2024), in some cases gabapentin (Chogtu et al., 2011), pregabalin (Hahm et al., 2012), lamotrigine (Chogtu et al., 2011) or baclofen (Deseure and Guy, 2017) are used as reference compounds. Although topiramate is also used as an adjuvant analgesic in trigeminal neuralgia, few clinical data are available regarding its efficacy, and it has not been investigated in the CFA-induced animal model of inflammatory orofacial pain.

In this study we aimed to pharmacologically validate the CFA-induced orofacial allodynia model using topiramate in comparison with the gold standard antimigraine serotonin 5-HT1B/D receptor agonist drug sumatriptan (Tfelt-Hansen and Hougaard, 2013). Topiramate was developed originally to treat epileptic seizures, however, it is also indicated to prevent migraine attacks and neuralgia related to trigeminal activation (Raffaelli et al., 2023). It mainly inhibits the voltage-gated Na^+^ and Ca^2+^ channels, however it can modulate the function of several other targets such as the GABA_A_ receptor, glutamate receptors, K^+^ channels (Raffaelli et al., 2023). Topiramate also proved to be effective in neuropathic and mediator-based pain-related animal models (Bischofs et al., 2004; Wieczorkiewicz-Plaza et al., 2004; Lopes et al., 2009; Paranos et al., 2013; Pradhan et al., 2014). Furthermore it has been described that topiramate inhibits microglial activation and thus inflammatory mediator release [e.g., tumor necrosis factor-α, interleukin-1β, interleukin-6) (Su et al., 2020; Faustmann et al., 2022)], therefore in this study we aimed to reverse the CFA-induced inflammatory orofacial allodynia with topiramate. Although the CFA-induced inflammatory orofacial pain is not migraine model, its pathophysiological mechanisms are closely related to headache and migraine, therefore sumatriptan, the antimigraine agent which inhibits trigeminal activation, may also be effective in this model.

Our results showed that topiramate reversed chronic CFA-induced inflammatory orofacial allodynia in rats, therefore, this model could be validated with this adjuvant analgesic. These results help to identify novel therapeutic options against orofacial pain-related syndromes including potential repurposing of topiramate for this indication.

## 2 Materials and methods

### 2.1 Animals, drugs, and chemical

The experiments were performed on 120 male Sprague-Dawley (SPRD) rats (Envigo, Akronom Ltd., Hungary) weighing 250–300 g. They were housed in the animal house of the Department of Pharmacology and Pharmacotherapy (University of Pécs) under standard light-dark cycle (12-h light/dark cycle) and temperature (24°C–25°C) conditions, food and water were provided *ad libitum*. The study was accomplished in accordance with the Ethical Codex of Animal Experiments of the University of Pécs and the 1998/XXVIII Act of the Hungarian Parliament on Animal Protection and Consideration Decree of Scientific Procedures of Animal Experiments (243/1988), and approved by the Ethics Committee on Animal Research of Pécs University and the license was given (license No.: BA02/2000–75/2023.).

CFA was purchased from Merck Life Science Ltd. (Budapest, Hungary). Topiramate was obtained from Thermo Fisher Scientific (Waltham, Massachusetts, United States), while sumatriptan from Biosynth (Louisville, KY, United States). Topiramate and sumatriptan were dissolved in saline containing 5% Tween 80 before the administrations. Pentobarbital was obtained from Alfasan International B.V. (Euthanimal 40% ad. us. vet., Alfasan International B. V., Woerden, Netherlands).

### 2.2 Experimental protocol and functional measurements

**FIGURE 1:**
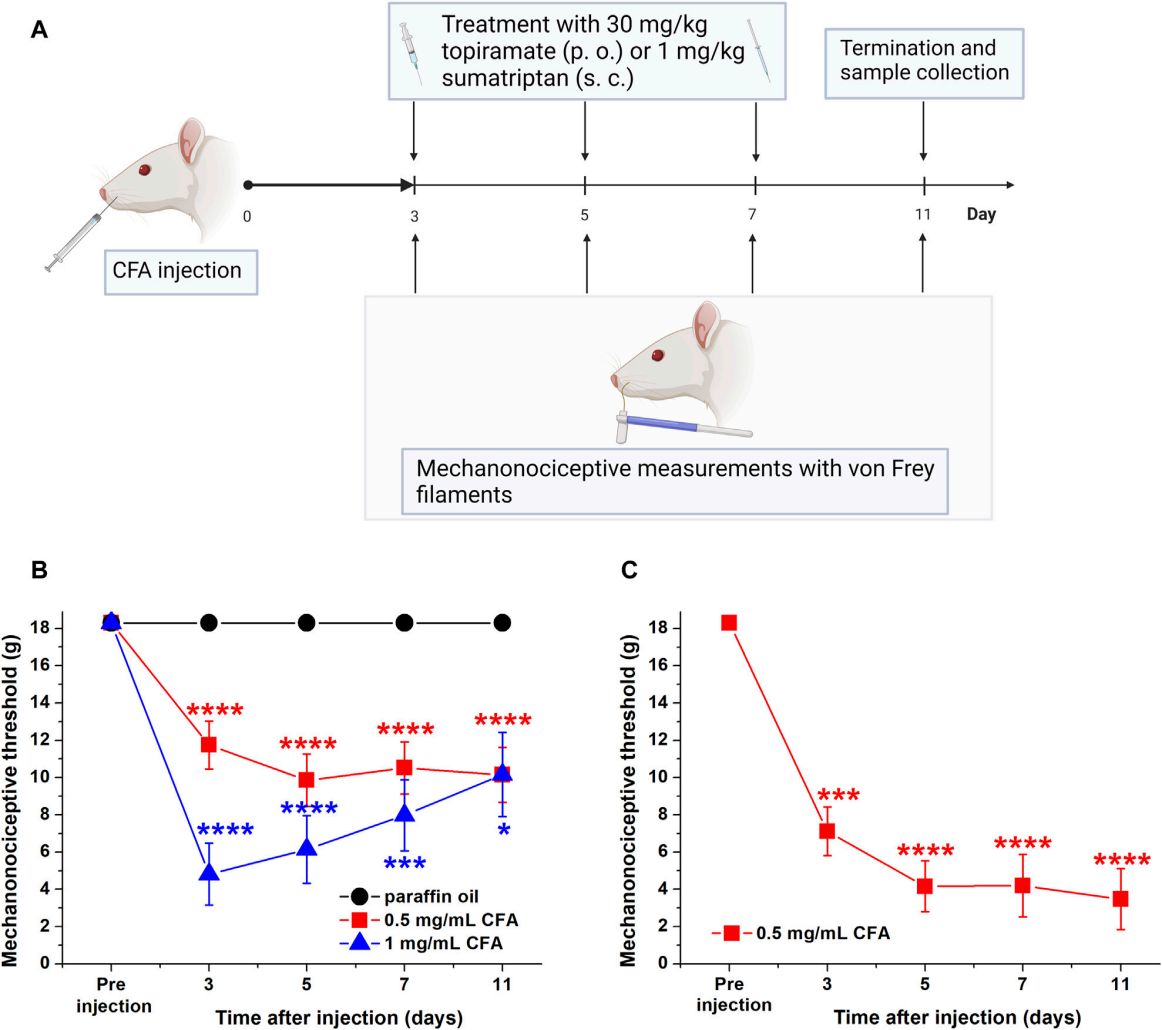
**(A)** Schematic representation of the experimental paradigm. **(B)** Mechanonociceptive threshold values after 0.5 mg/mL and 1 mg/mL CFA injection into the whisker pad in comparison with the control paraffin oil. Data points represent the means + SEM of n = 10 (paraffin oil); n = 29 (0.5 mg/mL CFA); n = 14 (1 mg/mL CFA), as analyzed by repeated measures one-way ANOVA followed by Dunnet’s post-test (*p ≤ 0.05, **p ≤ 0.01, ***p ≤ 0.001, ****p ≤ 0.0001 vs pre-intervention data). **(C)** Mechanonociceptive threshold values induced by 0.5 mg/mL CFA injection into the whisker pad of selected animals in which allodynia developed (threshold <18.30 g). Data points represent the means + SEM of n = 17 rats (*p ≤ 0.05, **p ≤ 0.01, ***p ≤ 0.001, ****p ≤ 0.0001 vs pre-intervention values, as analyzed by repeated measures one-way ANOVA followed by Dunnet’s post-test).

Experiments were performed as illustrated in Figure 1A. Briefly, chronic orofacial inflammation was induced by subcutaneous (s.c.) injection of CFA (killed mycobacteria suspended in paraffin oil; 50 μL, one or 0.5 mg/mL) into the right whisker pad under 50 mg/kg intraperitoneal (i.p.) pentobarbital anaesthesia. Paraffin oil was injected the same way in control rats.

The mechanonociceptive threshold of the whisker pad was determined by von Frey filaments (Stoelting, Wood Dale, IL, United States) before CFA (or paraffin oil) injection and after on days 3, 5, 7, and 11. The animals were habituated to the filaments after which baseline measurements were performed to exclude rats which produced basically lower mechanical threshold values (<10 g). During the measurements animals were lightly restrained by the experimenter using a cotton glove and after the animal calmed down, the von Frey filaments (15.14, 8.51, 5.50, 3.63, 2.04, 1.20, 0.69, 0.41 g) were applied using the “up-and-down” method (Chaplan et al., 1994): testing was started in the middle of the series using the 4.31 marked filament (2.04 g), and when response was positive, the next weaker filament was used, however in the case of a negative response, the next stronger filament was applied. Each filament was applied on the whisker pad 3 times in succession and if the animal showed at least twice avoidance behavior (withdrawal response) the response was positive, otherwise the answer was negative. Withdrawal response was considered in cases of face stroking with the forepaw, retreating from the stimulus, face shaking, vocalization, enhanced face grooming. Allodynia (decrease of the touch sensitivity thresholds) was expressed as the 50% orofacial withdrawal threshold.

### 2.3 Drug treatments

The effects of topiramate (30 mg/kg per os (p.o.) 0.5 mL/100 g) and sumatriptan (1 mg/kg s. c., 0.25 mL/100 g) were tested on days 3, 5, and 7 after CFA injection (Figure 1A). The first control measurement was performed before the treatments on day 3 and rats with thresholds below 18.3 g were selected and randomized in two different groups (drug- or vehicle treatments) for further p. o. (oral gavage in conscious animals) or s. c. treatments. This randomization remained during the whole experiment. We used 5% tween 80 solution as vehicle (which was the solvent for topiramate and sumatriptan). All treatments and measurements were performed in a double-blind manner. The effects of the drugs were investigated before drugs and 60, 120, and 180 min after the treatments each day (3, 5, and 7 days after CFA). We did not observe any changes in the behavior of the animals due to the repeated measurements that would affect the results, they did not show any escape or distress behavior in any case.

### 2.4 Evaluation of gene-expression changes in the TG samples

Ingenuity Pathway Analysis (IPA) was performed (software version 111725566 (QIAGEN) based on its manually curated Knowledge Base) on the transcriptomic data of the TG samples obtained from CFA-treated rats of the same protocol on day 7 (Aczél et al., 2018; Aczél et al., 2020). The microarray data analysis identified 512 differentially expressed (319 up- and 191 downregulated) transcripts between the contralateral (control) and ipsilateral (CFA-treated) TG samples at a statistically significant level (*p* ≤ 0.05), with fold change |FC|> 2. In this study, we reviewed the data and selected genes that may be involved in the mechanism of action of topiramate and sumatriptan. The expression changes for the selected genes between the two groups were visualized on a heat map (Figure 4), and hypothesis-driven exploration of the potential pathways (e.g., headache, pain, inflammation, and the tested drugs) was demonstrated (Figure 5).

### 2.5 Statistical analyses

All data are represented as the means of the individual threshold values with the standard errors of the mean (SEM) values, statistical analysis was performed by the GraphPad Prism nine software. Although not all data sets showed normal distribution in the present series of experiments with the Shapiro–Wilk test due to the relatively small group sizes and big variations, based on earlier results and literature data in the same model repeated measures two-way ANOVA followed by Šidák’s post-test were conducted to determine differences between measurements of the vehicle- and drug-treated groups. One-way repeated measures ANOVA followed by Dunnett’s post-test were conducted to test differences between pre dose and post dose measurements. Probability values *p* ≤ 0.05 were accepted as significant.

Effect size is a value measuring the strength of the relationship between two variables in a population, calculated by Hedge’s g, which is the estimated standardized difference between the means of two populations (effect size calculator: https://www.socscistatistics.com/effectsize/default3.aspx). Hedges’ g provides a correction for small-sample bias (Hedges, 1981; Hedges and Olkin, 2014; 1. Equation):

$$Hedges^{\prime }g=\frac{M_{1}-M_{2}}{{SD}_{pooled}^{*}}$$

Where.

$M_{1}-M_{2}$ = difference in means

${SD}_{pooled}^{*}$ = pooled and weighted standard deviation (2. Equation)

$${SD}_{pooled}^{*}=\sqrt{\frac{\left(n_{1}-1\right){SD}_{1}^{2}+\left(n_{2}-1\right){SD}_{2}^{2}}{n_{1}+n_{2}-2}}$$

## 3 Results

### 3.1 The 0.5 mg/mL CFA induces stable orofacial allodynia in rats: optimal for further drug testing

First, we tested the behavioural effects of 1 mg/mL and 0.5 mg/mL CFA to determine the optimal concentration for further drug effect analysis. Although the higher CFA concentration induced greater facial allodynia, much more stable touch sensitivity reduction was observed with the lower CFA concentration (Figure 1B). On day 3, the threshold values decreased from 18.30 g to approximately 11 g (low CFA) and 5 g (high CFA concentration), respectively, however a slight increase was observed in the case of higher CFA concentration (on days 5, 7, and 11). Animals in the paraffin oil-treated group did not show any change in the threshold values.

We also calculated the change in the threshold values by excluding the animals which did not developed allodynia (non-responsive rats: approximately 40% of the animals, n = 12/29). Significant decrease in mechanonociceptive thresholds was observed (from 18.30 g to approximately 7 g) 3 days after CFA injection, which became more robust on days 5, 7, and 11 (Figure 1C).

### 3.2 Topiramate blocks CFA-induced chronic orofacial allodynia after acute administration

**FIGURE 2:**
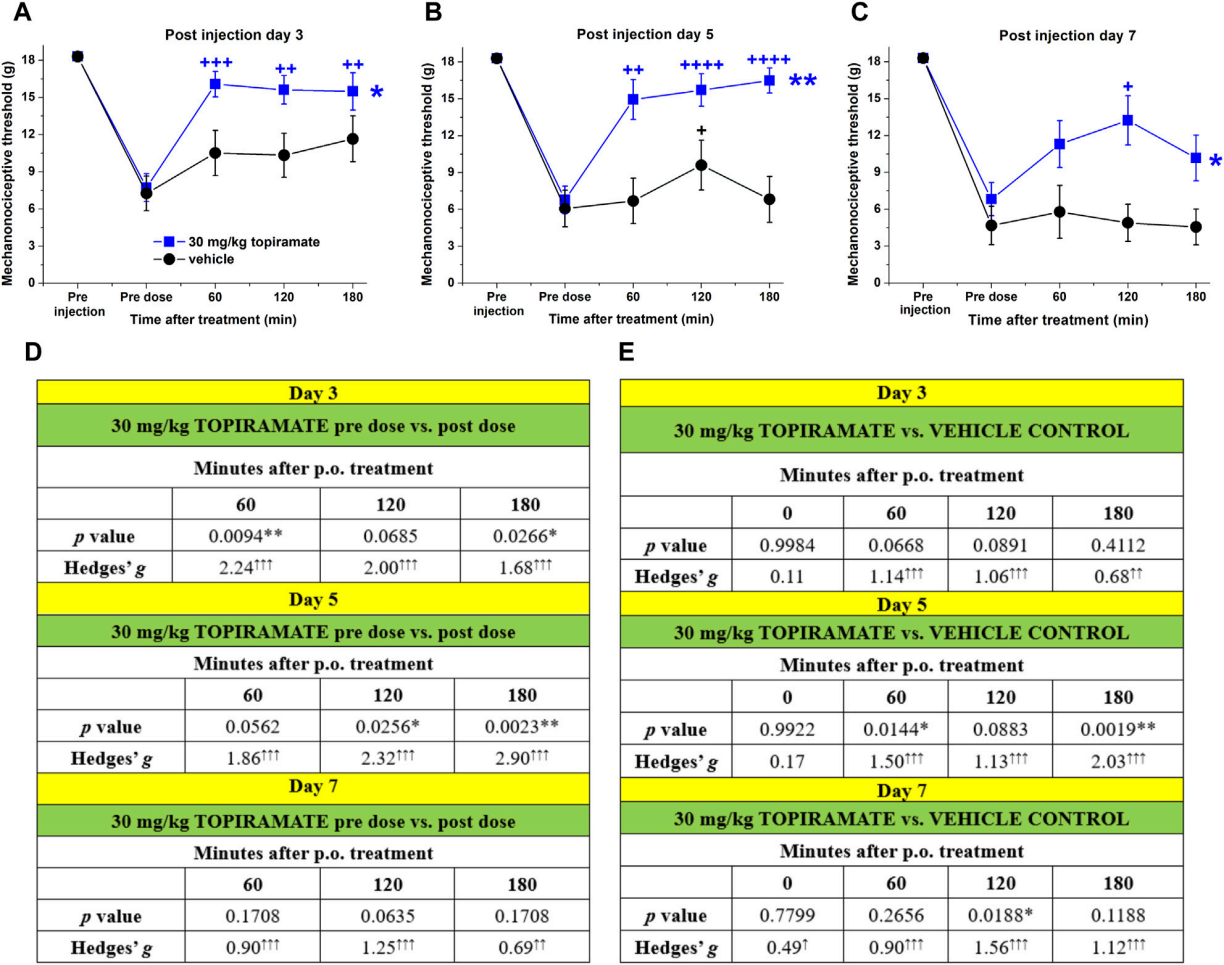
Effect of topiramate on the CFA-induced allodynia 3 **(A)**, 5 **(B)**, and 7 **(C)** days after the injection: before topiramate or vehicle (5% Tween 80 solution) treatment animals received 0.5 mg/mL CFA injection into the right whisker pad, then threshold values were investigated before (pre dose) and 60, 120, and 180 min after the treatments. Data points represent the means + SEM of n = 9–12 rats/group (the results are pooled from several individual cohort studies). Asterisks denote statistically significant differences between control and experimental groups as analyzed by repeated-measures two-way ANOVA (*p ≤ 0.05, **p ≤ 0.01). Crosses denote statistically significant differences between pre dose and post dose measurements within the same group (+*p* ≤ 0.05, ++*p* ≤ 0.01, +++*p* ≤ 0.001, ++++*p* ≤ 0.0001) as analyzed by repeated measures one-way ANOVA followed by Dunnet’s post-test. Effect size values and *p* values calculated between pre vs post dose thresholds **(D)** and topiramate vs vehicle treatment **(E)**. Effect size was calculated using Hedges’g, arrows denote the magnitude of the effect size and direction (↑small >0.2↑↑medium >0.5; ↑↑↑large >0.8).

In order to characterize the CFA-induced inflammatory, trigeminal-activated orofacial allodynia model the effect of topiramate on the mechanonociceptive thresholds was investigated in comparison with sumatriptan. The effect of 30 mg/kg topiramate was tested 3, 5, and 7 days after 0.5 mg/mL CFA injection. Topiramate showed a significant anti-allodynic effect comparing the pre dose and post dose of drug and comparing the vehicle-treated to the drug treated group (Figures 2A–C) supported by the medium and large effect size values, and the results of the repeated-measures one- and two-way ANOVA tests (Figures 2D, E).

### 3.3 Sumatriptan exerts mild inhibitory effect on CFA-induced chronic facial allodynia after acute administration

**FIGURE 3:**
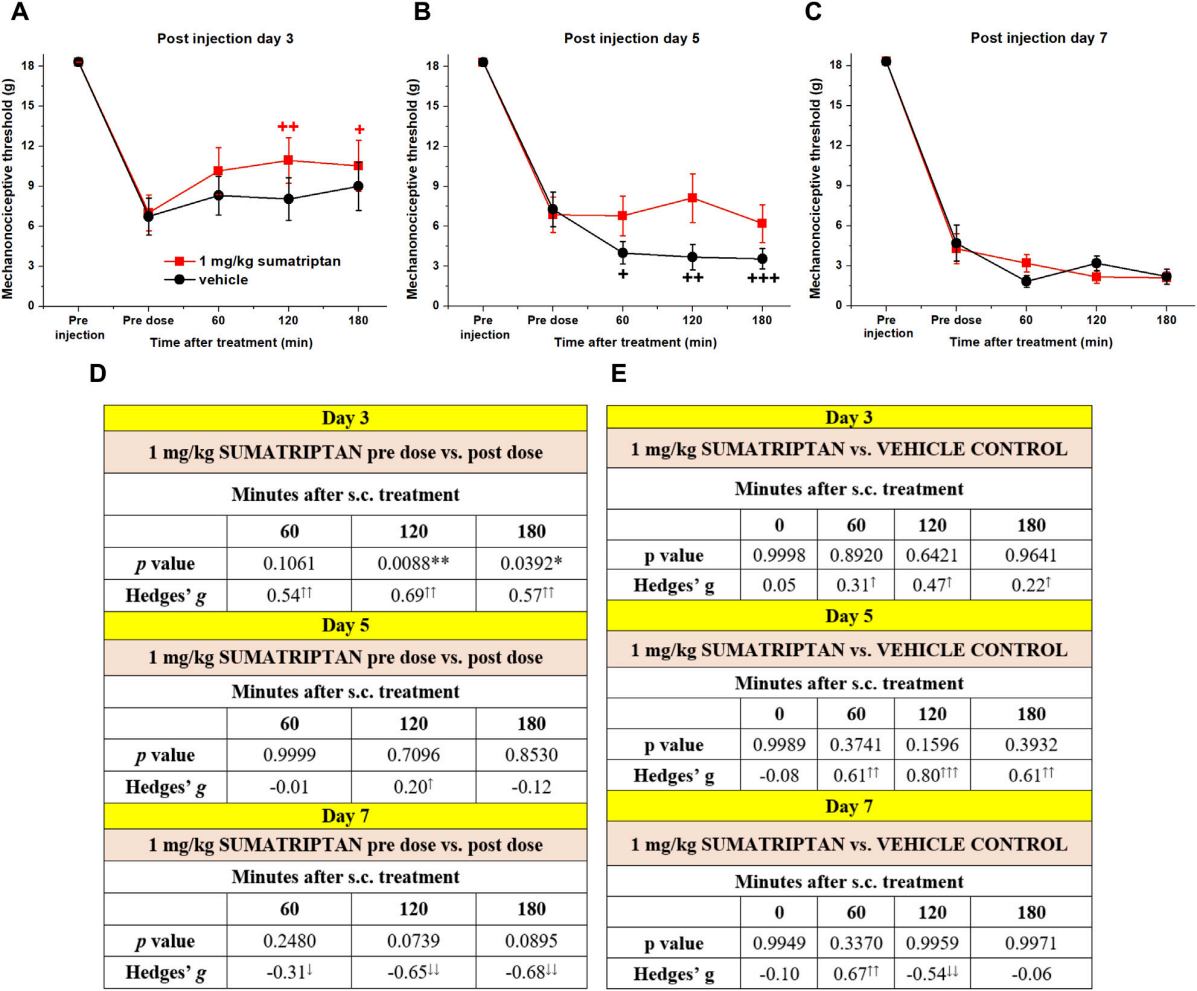
Effect of sumatriptan on orofacial allodynia after 0.5 mg/mL CFA injection into the right whisker pad 3 **(A)**, 5 **(B)**, and 7 **(C)** days after the induction of the inflammation. Mechanonociceptive threshold values were investigated before (pre dose) and 60, 120, and 180 min after sumatriptan (1 mg/kg) or vehicle (5% Tween 80 solution) treatment. Data points represent the means + SEM of n = 13–15 rats/group (the results are pooled from several individual cohort studies), analyzed by repeated measures two-way ANOVA compared to the vehicle treatment and by repeated measures one-way ANOVA followed by Dunnet’s post-test compared to the vehicle treatment. Effect size values and *p* values calculated between pre vs post dose thresholds **(D)** and sumatriptan vs vehicle treatment **(E)**. Effect size was calculated using Hedges’g, arrows denote the magnitude of the effect size and direction (↑small >0.2↑↑medium >0.5; ↑↑↑large >0.8).

The effects of lower (0.3 and 1 mg/kg) and higher (3 and 10 mg/kg) doses of sumatriptan were examined on orofacial allodynia provoked by 1 mg/mL CFA, however none of these doses applied were effective to reverse the developed robust allodynia (*see in* Supplementary Material: Supplementary Figure S1). Investigations were then repeated with 1 mg/kg sumatriptan, however in this case the lower CFA concentration was used to induce a lower degree of allodynia (Figures 3A–C). In the sumatriptan-treated group, sumatriptan caused a significant threshold increase compared to pre dose (day 3), which was also supported by the medium effect size values (Figures 3A, D). Furthermore, sumatriptan also induced a slight anti-allodynic effect compared to the vehicle-treated group (day 3 and 5), supported by the small, medium and large effect size values (Figure 3E), however repeated measures two-way ANOVA did not show significant differences between the two group. Furthermore, the significant difference between the treated and control groups on day five is due to the lower thresholds of the control animals after treatment (Figure 3B).

### 3.4 Potential mechanisms of action of topiramate and sumatriptan at the level of the primary sensory neurons in the TG: bioinformatic analysis

**FIGURE 4:**
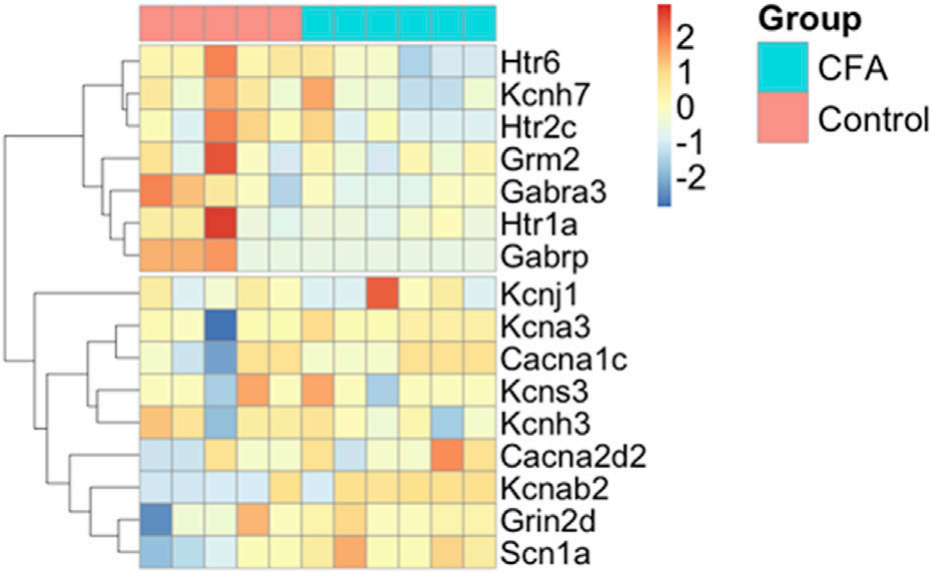
Change in the mRNA levels between CFA-treated (ipsilateral) and contralateral side (control group) in TG samples, 7 days after CFA injection. Orange color means high, while blue indicates low expression. The column represents the individual samples, while the row shows one differentially expressed (DE) feature (microarray feature identifiers). Data are obtained from the previous results of the research group.

**FIGURE 5:**
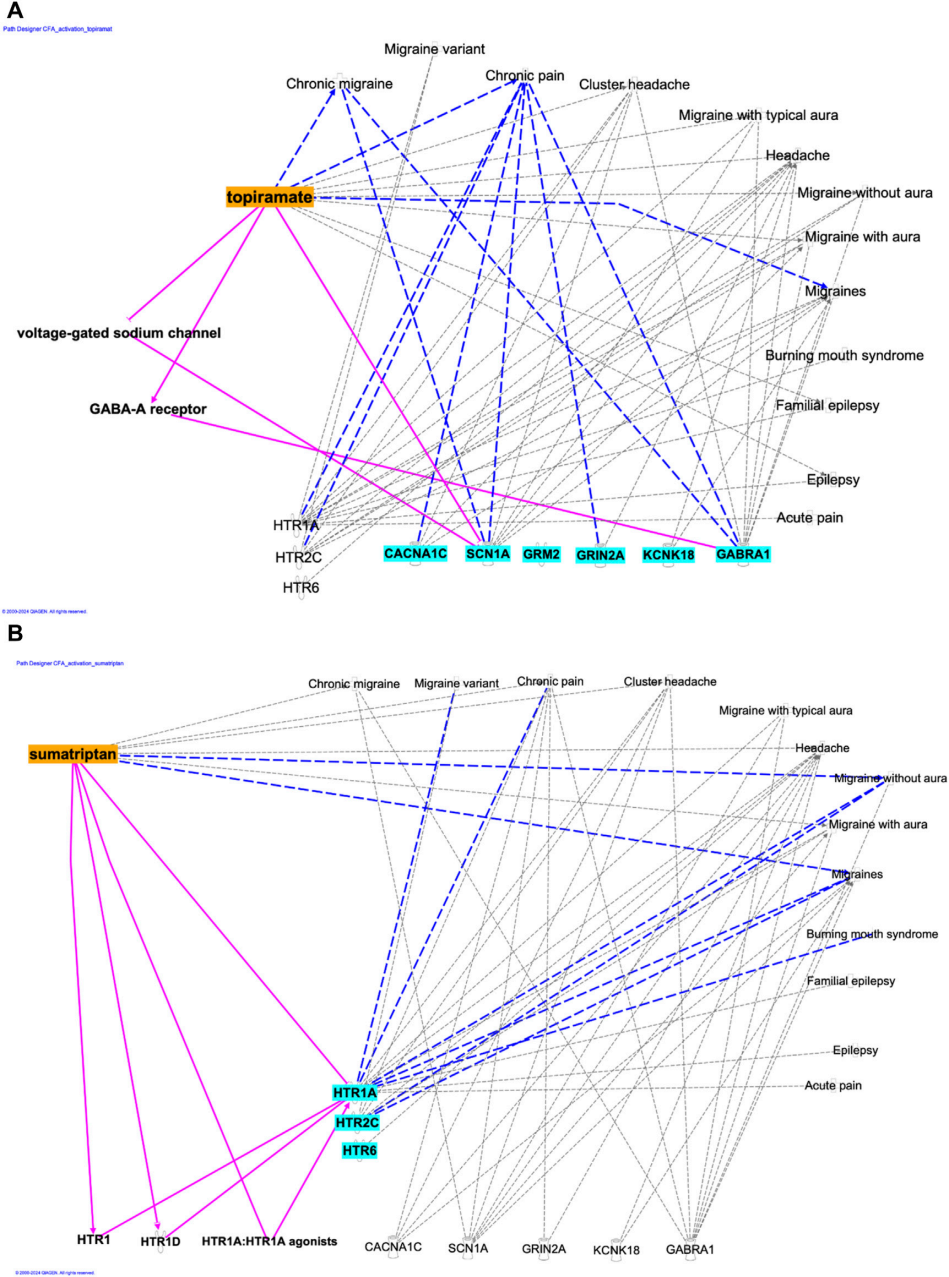
**(A)** The direct and indirect relationships between topiramate, selected genes, and neuro-inflammation-related terms. **(B)** The direct and indirect relationships between sumatriptan, selected genes, and neuro-inflammation-related terms.

Transcriptomic alterations in the primary sensory neurons of the TG might be associated with the molecular mechanisms of topiramate and sumatriptan. Figure 4 represents the fold change data obtained from our previous study of the same model (Aczél et al., 2018). Significant upregulation of Cacna1c, Scn1a, Grm2 Grin2d was detected on the CFA-treated ipsilateral side, of which Scn1a can be inhibited by topiramate according to the IPA analysis. On the other hand, CFA-treatment induced significant decrease in the expression of Htr2c, Htr6, Kcnj1, Kcnh3, Kcnh3, Kcns3, Kcnab2, Kcnh7, Gabra3, which encode proteins that may be activated by topiramate or sumatriptan. Topiramate can also exert direct effects not only on voltage-gated Na^+^ channels and GABA_A_ receptors, but also on Scn1a gene (Figure 5A), the expression of which was significantly increased after CFA treatment (Figure 4).

Regarding the mechanism of action of sumatriptan, the results showed a direct link between sumatriptan and the Htr1A gene (Figure 5B), the expression of which was significantly reduced after CFA treatment (Figure 4).

## 4 Discussion

We demonstrate here the first results on the acute anti-allodynic effect of topiramate in rat chronic orofacial inflammation which provides pharmacological validation of this model to test novel analgesic targets and candidates. Interestingly, the gold standard antimigraine drug sumatriptan also exerted some inhibitory effect after acute administration on day 3 and 5 suggesting common pathophysiological mechanisms of trigeminal activation related to chronic orofacial pain and migraine. Although carbamazepine is the first-line treatment for trigeminal neuralgia (Bendtsen et al., 2019; Clark et al., 2020; Gambeta et al., 2020), which is a common orofacial pain condition (Okeson and de Leeuw, 2011; Gambeta et al., 2020), topiramate has also been shown to have analgesic effects in clinical practice (Zvartau-Hind et al., 2000; Solaro et al., 2001; Domingues et al., 2007; Wang and Bai, 2011). Furthermore, the clinical efficacy of carbamazepine (Di Stefano et al., 2021; Guo et al., 2024) is also supported by preclinical animal data (Chogtu et al., 2011; Hahm et al., 2012; Pineda-Farias et al., 2021; Baggio et al., 2024; Song et al., 2024), however preclinical studies demonstrating the efficacy of topiramate treatment on the CFA-induced inflammatory orofacial pain are still lacking.

Inflammatory allodynia/hyperalgesia induced by CFA is a widely used experimental method (Takeda et al., 2008; Magni et al., 2015; Takeda et al., 2018; Lin et al., 2022). CFA is a water-in-oil emulsion containing *Mycobacterium tuberculosis*. *Mycobacterium* induces inflammation due to its ability to increase IL-12 production and activate toll-like receptors in dendritic cells (Rafiyan et al., 2023). CFA also induces the production of Th1 cytokines (e.g., IL-2, INF-γ, TNF, TNF-β), increases humoral and cellular immunity and mediates specific cellular immune responses (Huang et al., 2024). Furthermore, the CFA-induced peripheral inflammation can also provoke glial activation, leading to the enhanced expression of proinflammatory cytokines (IL-1b, IL-6 and TNF-α) in the central nervous system (Raghavendra et al., 2004).

Here, we present that about 60% of the CFA-treated animals showed stable allodynia, which is in good agreement with our previously published results (Aczél et al., 2018; Aczél et al., 2020). Studies based on CFA use frequently undiluted form of the adjuvant to induce local inflammation (Yaster et al., 2011; Ye et al., 2018; Ferdousi et al., 2023), which is why we also first used the concentrated emulsion in our experiments. However, the results showed that concentrated CFA (1 mg/mL) induces exceedingly robust allodynia in the orofacial region, which could not be reversed pharmacologically even with high drug doses (Figure 1A; Supplementary Figure S1). This also provides crucial information for future use of this model in drug testing.

Topiramate significantly reduced CFA-induced inflammatory allodynia on day 3, which is in good agreement with the acute analgesic effect of the drug observed in other allodynia models such as the nitroglycerin-induced model, chronic constriction injury, formalin-induced hyperalgesia, carrageenan-induced inflammation (Bischofs et al., 2004; Lopes et al., 2009; Paranos et al., 2013; Pradhan et al., 2014). Chronic administration of topiramate exerted significant anti-allodynic effect in nerve-ligation and mediator-based nitroglycerin-induced pain models (Wieczorkiewicz-Plaza et al., 2004; Pradhan et al., 2014). Literature data suggest that topiramate can inhibit microglial activation and thus the release of inflammatory mediators (e.g., tumor necrosis factor-α, interleukin-1β, interleukin-6) (Su et al., 2020; Faustmann et al., 2022)), this may explain the anti-allodynic effect of topiramate in the CFA-induced inflammatory orofacial allodynia model. The efficacy of topiramate in this model may also be explained by the gene expression changes measured in TG tissues of CFA-treated rats (Aczél et al., 2018). We identified several differentially expressed genes which might be responsible for the expression of proteins involved in the mechanism of action of topiramate (e.g., Scn1a, Cacna1c). This is supported by the IPA analysis suggesting several relationships between topiramate, the differentially expressed genes in the TG and neuroinflammation-related mechanisms (Figure 5A). Furthermore, it has been reported that topiramate can reduce excitatory neurotransmitter release (Holland et al., 2010; Silberstein, 2017) by binding to the voltage-gated Na^+^ channels as well as kainite and AMPA receptors of glutamate, resulting in the downregulation of these structures (Mula et al., 2006; Raffaelli et al., 2023). Topiramate also inhibits voltage-gated Ca^2+^ channels on trigeminal nerve endings, which suggests its ability to prevent the release of vasoactive peptides, such as calcitonin gene-related peptide (Hoffmann et al., 2014; Raffaelli et al., 2023). Moreover, topiramate can also increase the inhibitory GABA activity, by upregulating the GABA_A_ receptors on neurons (Mula et al., 2006; Holland et al., 2010; Silberstein, 2017; Raffaelli et al., 2023) and alter the activation and sensitization of neurons, which may explain its preventive effect on migraine and cortical spreading depression (Ayata et al., 2006). All these mechanisms may play a role in its anti-allodynic effect in our model.

Interestingly, sumatriptan also showed a mild effect on CFA-induced inflammatory orofacial allodynia, suggesting that the mechanisms involved in orofacial pain may overlap with migraine disorders. This is consistent with other studies, in which sumatriptan significantly reduced allodynia in nerve ligation-induced chronic neuropathy models and was able to acutely reverse nitroglycerin-induced hyperalgesia as well as the neurogenic inflammation induced by the temporomandibular joint injection of CFA (Kayser et al., 2002; Oshinsky et al., 2012; Tomic et al., 2015; Lacković et al., 2016; Afshari et al., 2021). However, in contrast with our findings sumatriptan did not show any effect on the CFA-induced plantar hyperalgesia in rats (Pradhan et al., 2014). Mild effect of sumatriptan exerted in this model might be explained by gene expression changes in the TG of CFA-treated rats (Aczél et al., 2018). We found significantly lower expression of serotonin receptors encoding genes (Htr6, Htr2c, Htr1a) in the CFA-treated group, of which a direct association between htr1a gene and sumatriptan was identified by IPA analysis (Figure 5B).

In conclusion, CFA-induced chronic orofacial allodynia was significantly reduced by topiramate, therefore this model is suitable for testing novel drug candidates. Furthermore, our results also highlight the potential for repurposing topiramate for this condition.

## Data Availability

The raw data for the article are publicly available in the Zenodo repository, DOI 10.5281/zenodo.13283994 (https://zenodo.org/records/13283995). These data include the results of the von Frey measurements and the data obtained from the IPA analysis.

## Abbreviations

CFA, Complete Freund’s Adjuvant, IPA, Ingenuity Pathway Analysis; i. p.: intraperitoneal; p. o., per os; SPRD, Sprague-Dawley; s. c., subcutaneous; TG, trigeminal ganglion; TNC, trigeminal nucleus caudalis.
