# Supplementary material for: Topiramate inhibits adjuvant-induced chronic orofacial inflammatory allodynia in the rat
Source: Front Pharmacol. 2024 Aug 16;15:1461355. doi: 10.3389/fphar.2024.1461355 (PMC11361966; doi:10.3389/fphar.2024.1461355)
Supplement: Supplementary file 1 [file Image1.pdf]

Supplementary Material

1 Supplementary Figure

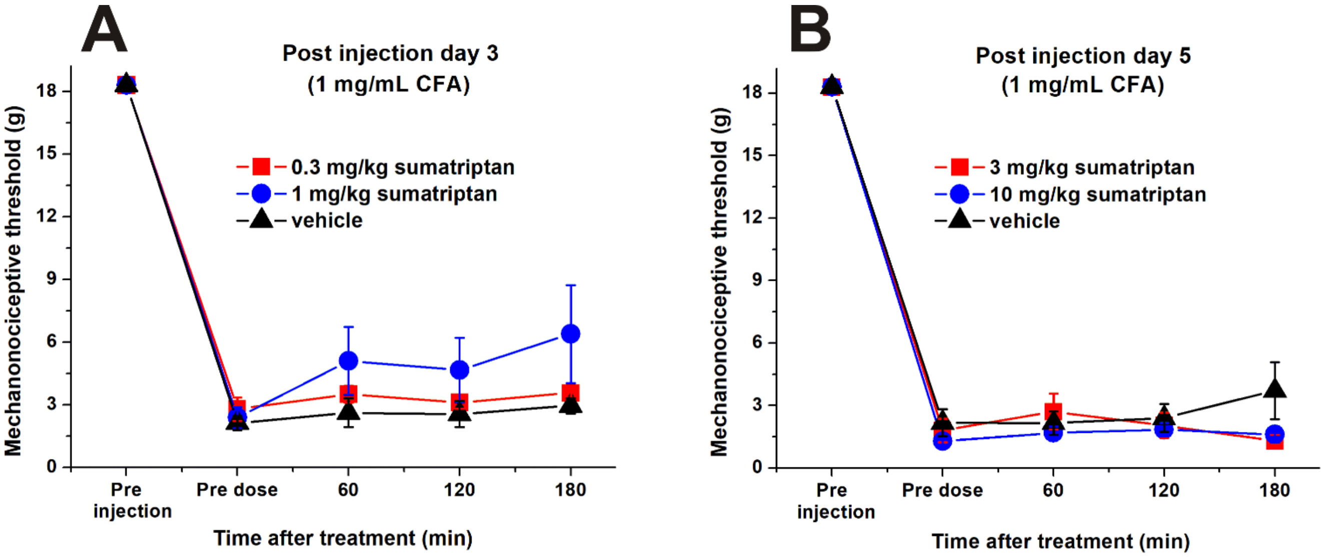

Supplementary Figure 1. Effect of sumatriptan on orofacial allodynia after 1 mg/mL CFA injection into the right whisker pad 3 **(A)** and 5 **(B)** days after the induction of the inflammation. Mechanonociceptive threshold values were investigated before (pre dose) and 60, 120, and 180 minutes after sumatriptan (0.3, 1, 3, 10 mg/kg) or vehicle (5% Tween 80 solution) treatment. Data points represent the means+SEM of n=5/group, analyzed by repeated measures two-way ANOVA compared to the vehicle treatment and by repeated measures one-way ANOVA followed by Dunnet's post-test compared to the vehicle treatment
